# *Helicobacter pylori* infection and pediatric asthma

**Published:** 2013-06

**Authors:** Abdullah Karimi, Koroush Fakhimi-Derakhshan, Farid Imanzadeh, Mohamad Rezaei, Zahra Cavoshzadeh, Saeid Maham

**Affiliations:** 1 Pediatric Infections Disease Research Center, Shahid Beheshti University of Medical Sciences, Tehran, Iran; 2 School of Medicine, Shahid Beheshti University of Medical Sciences, Tehran, Iran; 3 Departments of Pediatric Gastroenterology, Mofid Children's Hospital, Shahid Beheshti University of Medical Sciences, Iran

**Keywords:** *Helicobacter pylori*, urea breath test, asthma

## Abstract

**Objective:**

Childhood infectious diseases are one of the most known environmental pathogenic causes of childhood asthma. The high prevalence of both *Helicobacter pylori* infection and asthma in our country prompted us to assess anyprobable association between them in childhood.

**Methods:**

This cross-sectional study recruited 196 children aged 6 to 12 years old comprising 98 asthmatic (case group) and 98 healthy (control group) individuals. Urea breath test was performed for all of the children and *H. pylori* infection was compared between the two groups according to the urea breath test results.

**Results:**

Urea breath test was positive in 18 asthmatic (18.36) and 23 (23.36) healthy subjects but was not significantly different between the case and controls (p = 0.380). Further analysis in the asthmatic group revealed association of *H. pylori* infection withage (p < 0.001) and duration of asthma (p = 0.010). However, no significant correlation was found between sex, severity of asthma, controled asthma or abnormal pulmonary function tests with *H. pylori* infection (p= 0.804, 0.512, 0.854 and 0.292, respectively).

**Conclusion:**

Given the results of the study, *H. pylori* infection was not significantly different between asthmatic and healthy children. In asthmatic patients, there was no significant association between *H. pylori* infection and sex, severity of disease, control status of disease and normal or abnormal pulmonary function tests. *H. Pylori* infection had a significant association with increasing age and duration of asthma.

## INTRODUCTION

*Helicobacter pylori* infection is still one of the most common human infectious diseases worldwide and some recent serologic studies illustrated that most carriers of *H. pylori* have infected during childhood (1, 2). Developing and popular countries are more susceptible to this infection than others. *H. pylori* infection occurs globally with a prevalence of 20% to 50% in industrial countries and as high as 80% in developing countries (3). Shortly after discovery of *H. pylori* by Warren and Marshal (4), more pathological investigations illustrated the principal causative role of *H. pylory* in progression of a variety of chronic states (3, 5–8). *H. pylori* is a Gram-negative micro-aerophilic spiral bacterium that colonizes the gastric mucosal layer and causes a variety of diseases such as gastritis, peptic ulcer diseases, gastric cancers, and lymphoid tissue lymphomas (5–7, 9–11). However, associations of *H. pylori* infection with extra-gastric diseases such as respiratory diseases are still not clear and are under investigation by many scientists. Like many people in developing countries, Iranians also suffer from a high prevalence of *H. pylori* infection in addition to other diseases such as childhood asthma and allergy. Therefore, we conducted the present study to evaluate any possible association between *H. pylori* infection and childhood asthma using the urea breath test (UBT).

## MATERIALS AND METHODS

### Study population

During this cross-sectional study from January 2010 to March 2011, 196 children comprising 98 asthmatic patients who referred to our pediatric clinic of asthma and allergy (case group) and 98 healthy individuals (control group) were enrolled in the analysis. In the case group, documented asthmatic patients younger than 18 years were included. Patients with documented *H. pylory* infection, history of gastrointestinal diseases or proton-pump inhibitor use were excluded from the study. In the control group, all healthy staff's children younger than 18 years old were enrolled in the study (matched according to the age with case groups). The study was approved by ethic committee of the University and written informed consent was obtained from the parents.

UBT was performed for all subjects and the results in addition to the baseline characteristics were compared between the two groups. In the case group, UBT results were evaluated according to the age, sex, duration of asthma (years), severity and control status of the asthma and pulmonary functional test (PFT). Severity of the disease classified as mild/intermittent or more severe. PFT results classified as normal or obstructive disease.

### Statistical analysis

Data are reported as mean ± standard deviation (SD) otherwise as median and range. Independent sample T-test and chi-square were carried out when data were normally distributed. A p-value of less than 0.05 was defined as statistical significant. All analyses were performed using SPSS for Windows version 16.0.

## RESULTS

A total of 196 children aged 6 to 12 years old comprising 98 asthmatic and 98 healthy ones were studied. Baseline characteristics are shown in Table 1. UBT was positive in 18 of 98 asthmatic patients (18.36%) and 23 of 98 healthy individuals (23.46%). The test was not significantly different between the two groups (p= 0.380).

**Table 1:** Comparison of baseline characteristics between the two groups.

Characteristic	Patients group n = 98	Control group n = 98	P-value
Age(years)	8.64±2.04	8.43±2.04	
Gender			
Male	57 (58.16%)	60 (61.22%)	
Female	41 (41.84%)	38 (38.78%)	
Positive UBT	18(18.36%)	23(23.46%)	0.380

Data are presented as n(%) or mean ± standard deviation (SD). UBT; urea breath test

In the asthmatic group, UBT was evaluated according to the age, sex, duration of asthma (years), severity and control status of the disease and PFT (Table 2). It showed the test just was significantly different among the asthmatic patients according to the age and the duration of the disease P < 0.001 and P = 0.01, respectively.

**Table 2:** Urea breath test results among asthmatic patient.

Characteristic	Positive UBT n = 18	Negative UBT n = 80	P-value
Age (years)	10.39±1.33	8.25±1.96	<0.001
Gender			
Male	10	47	0.804
Female	8	33	
Duration of disease(years)	3.15±1.67	2.05±1.61	0.010
Severity of disease			
Persistent mild	11	47	0.854
Moderate and severe	7	33	
Control status of disease			
Control	16	64	0.512
Uncontrolled	2	16	
Pulmonary function tests			
Normal result	17	71	0.292
Obstructive disease	1	14	

Data are presented as n (%) or mean ± standard deviation (SD). UBT; urea breath test

## DISCUSSION

To the best of our knowledge, this is the first study to evaluate any possible relationship between *H. pylori* infection and childhood asthma using the UBT in our population. Given the results of the study, there was no significant difference between *H. pylori* infection and childhood asthma. But, we found an association between increased age and prolonged duration of disease in asthmatic children with more incidences of *H. pylori* infection.

Although, indistinct results of previous studies regarding correlation between *H. pylori* infection and asthma make comparison difficult; our results are some how in line with some epidemiological studies that believed *H. pylori* seropositivity is more prevalent in atopic diseases such as asthma, rhinitis and eczema (12–14). Although it is in contrast with some other studies that propose inverse association between asthma and *H. pylori* infection (15–17).

These differences may mostly be based on the diversity of study populations. In contrast to developing countries where the prevalence of both asthma and *H. pylori* infection is increasing in industrial countries, the incidence of *H. pylori* infection has had a decreasing trend contrary to the prevalence of childhood asthma which has increased over the recent decades (3, 18–20). This proposes that lack of early exposure to *H. pylori* may be an important indicator of development of pediatric asthma (15, 16). Chen *et al*. (16) published a study regarding the presence of an inverse association between *H. pylori* positivity and asthma in children. Their results also suggested that in the presence of allergic rhinitis this inverse association is even stronger than in the absence of it. Zevit *et al*, (17) also revealed that there is an inverse association between *H. pylori* seropositivity and pediatric asthma.

However we found no significant association, but concur with some previous reports such as Raj *et al*, (14) that suggested *H. pylori* has some sort of association with asthma however may not be directly involved in the initiation of it (14, 21). Raj *et al*. (14) proposed that *H. pylori* infection possibly will be an only indicator of poor hygiene and the poor hygiene through the hygiene theory of more exposure to the infectious agents, results in a low prevalence of asthma.

Umetsu and DeKruyffas well stated that *H. pylori* may indirectly induce asthma and allergic diseases through exaggerated T-helper2-biased (Th2-biased) response (21). Th2- immune responses itself are controlled by regulatory T-cells and it is hypothesized that *H. pylori*-specific CD4+ T-cells are accumulated in the infected gastric mucosa by *H. pylori* and could lead to Th2-biased immune responses that initiate the development of asthma and allergic diseases (21–23).

On the other hand, study that was conducted on 2437 adults aged 18-70 years by Fullerton *et al*, (24), failed to find any association between positive *H. pylori* infection and either COPD, asthma, allergic diseases, atopic diseases or abnormal PFT. Furthermore, results from a community-based study of young adults provided no evidence that seropositive *H. pylori* patients had a lower level of IgE sensitization (25). These inconsistent findings of different investigations may be due to the regional variations in the epidemiology of the infection, as well as the different socioeconomic and living status in different countries.

However, due to the wide discrepancies reported in this regard further prospective longitudinal studies are needed to provide additional information to clarify the precise relationship between *H. pylori* infection and asthma in children from both developed and developing countries.

In conclusion, the results showed no association between the childhood asthma and the *H. pylori* infection. Although, we found that increased age and prolonged duration of the disease were associated with more *H. pylori* infection prevalence in the asthmatic population.
